# Spatial monitoring technologies for coupling the soil plant water animal nexus

**DOI:** 10.1038/s41598-022-07366-2

**Published:** 2022-03-03

**Authors:** Amanda J. Ashworth, Tulsi Kharel, Tom Sauer, Taylor C. Adams, Dirk Philipp, Andrew L. Thomas, Phillip R. Owens

**Affiliations:** 1 Poultry Production and Product Safety Research Unit, USDA-ARS, 1260 W Maple St, Fayetteville, AR 72701 USA; 2 Crop Production Systems Research Unit, USDA-ARS, 141 Experiment Station Road, Stoneville, MS 38776 USA; 3 National Laboratory for Agriculture and the Environment, USDA-ARS, 1015 N University Blvd, Ames, IA 50011 USA; 4 Animal Science Department, University of Arkansas, 1120 W Maple St, Fayetteville, AR 72701 USA; 5 Division of Plant Sciences, Southwest Research Center, University of Missouri, 14548 State Rd H, Mt. Vernon, MO 65712 USA; 6 Dale Bumpers Small Farms Research Center, USDA-ARS, 6883 Hwy 23, Booneville, AR 72927 USA

**Keywords:** Plant sciences, Systems biology, Environmental sciences

## Abstract

Systems-level studies aimed at determining how soil properties are linked to plant production and ultimately animal response spatially are lacking. This study aims to identify if grazing pressure is linked to soil properties, terrain attributes, and above-ground plant accumulation and nutritive value in a silvopastoral (or integrated tree-livestock) system. Overall, cattle prefer grazing native grasses (2.81 vs. 1.24 h ha^−1^ AU^−1^) and udic (dry) landscape positions compared to aquic (wet) areas (2.07 vs. 1.60 h ha^−1^ AU^−1^). Greater grazing frequency occurs in udic soils with greater phosphorus and potassium contents and with accumulated forage with less lignin (*P* ≤ 0.05), which correspond to reduced elevation and greater tree height and diameter (shade) during summer mob grazing. Combining spatial monitoring technologies (both soil and animal) with forage allowance can optimize grazing systems management and sustainability spatially and temporally.

## Introduction

Protein demands are increasing worldwide, as is the need for enhanced efficiency of livestock production. Improved understanding of linkages between animal behavior and environmental interactions will be required to optimize management of the livestock-grazing environment. However, technologies and methodologies that monitor and develop linkages among soil properties, forage growth, and ultimately preferential grazing based on landscape attributes are very limited. Therefore, to maximize grazing efficiencies in animal production, systems-level assessments of soils, plants, and animals are needed.

Silvopastures, or the integration of trees and animal production, are beneficial to soil quality due to manure deposition and can increase land equivalent ratios, carbon (C) sequestration, and ecosystem services, such as reduced cattle heat stress relative to monocropping systems^1–3^. In addition, nutrient depositions from animal excreta or external additions such as poultry litter may further increase below- and above-ground plant growth and quality^4^. However, correlations between forage quality and grazing patterns are limited, particularly for grasslands, the largest land-use category in the US^5^.

The introduction of tracking receivers in 1989^6^ and the more recent addition of commercial Global Navigation Satellite System (GNSS) collars, has dramatically improved the ability to study animal grazing preference, movement, and environmental interactions^7,8^. In tandem, digital soil mapping (DSM) procedures have made drastic advancements from thematic soil maps^9^. Landscapes can now be classified into homogenous terrain units^9,10^, and soil attributes in each unit can then be grouped by soil functional similarities^11^. Such a classification of continuous terrain can be done following fuzzy logic^12^ and clustering algorithms^13^. Variation in topography is now studied using terrain attributes, which can be derived from a digital elevation model (DEM) through digital terrain analysis^14^. Novel approaches in DSM and geospatial techniques help identify spatial relationships between soils and terrain attributes^15,16^, but this approach has not been integrated with GNSS collar-based information.

In efforts to improve pasture management and utilization, changes to cattle behavior based on forage allowance were determined using GNSS technology and Normalized Difference Vegetation Index (NDVI)^17^. In this experiment, cattle grazing reduced when NDVI declined, indicating these technologies may aid in the monitoring of sustainable grazing. In a study by ref.^18^, global positioning system (GPS) collars were used to track two cattle breeds in a range system and found that one breed favored forage species with higher nutritive values, with grazing locations corresponding to distribution patterns known to negatively impact desirable natural resources. Neither study evaluated grazing pressure in relation to topography.

Topography plays a crucial role in spatial distribution of available soil water, which in turn may influence nutrients, and ultimately forage production and animal grazing. Therefore, this study aims to evaluate: (1) causative factors of preferential grazing in a silvopastoral system by assessing soil physiochemical properties and terrain attributes by tracking grazing patterns of cattle using GPS collars; (2) grazing preference based on forage species, soil moisture regimes, and soil fertility; and, (3) grazing linkages with forage mass accumulation and nutritive value.

Prior to administering sustainable grazing lands management strategies, forage mass, accumulation, nutritive quality, terrain attributes, and soil parameter linkages with animal response must be known. Pasture availability, spatially and temporally, is one of the least understood variables affecting animal production, efficiency, and use of pasture by grazing livestock. In this systems experiment, cattle are 127% more likely to graze the native grass mix than the C_3_ species, orchardgrass, likely owing to the C_3_ species increasing in maturity and decreasing in palatability. Consequently, cattle spent more grazing hours foraging the native grass species under the drier soil moisture conditions, particularly as the season progressed. In this study, cattle grazing frequency vary spatially, temporally, and annually, with a lower number of animal hours spent in wetter, deeper soils across all years. Soil moisture regime patterns are consistent across years, with distinct landscape-based patterns; therefore, grazing distribution and frequency is linked to the grazing environment and ultimately terrain attributes. Overall, topography parameters help explain soil properties—plant growth (tree and forages)—and ultimately animal grazing pressure across this continuum.

## Results

### Temporal primary productivity dynamics based on forage species and fertility

The three-way (forage species × poultry litter fertility treatment × forage sampling date) interaction did not affect forage mass or biomass accumulation (*P* ≥ 0.05). However, there was a two-way interaction (*P* ≤ 0.05) between forage species (C_3,_ orchardgrass and C_4,_ mixed native) and fertility, forage species and sampling date (mid-May, late-May, early- June, mid-June, and late-June), and sampling date, and fertility for biomass accumulation. For forage mass, only a forage species × fertility interaction occurred (*P* ≤ 0.05). Accumulated biomass yields of fertilized orchardgrass were greater (*P* ≤ 0.05) than that of unfertilized orchardgrass and the fertilized or non-fertilized native grass mix.

There were no two-way interactions (*P* ≥ 0.05) among forage species × fertility treatment for either forage mass or biomass accumulation nutritive parameters, excluding N removal and P and S forage tissue content during biomass accumulation harvests (across all harvest dates). Poultry litter applications for both forages resulted in the greatest N removal and P content, with S content being greatest for orchardgrass during biomass accumulation harvests (Table 1).

**Table 1:** Forage yield and composition based on species and soil fertility.

Species	Fertility	Forage yield	N removal	C^b^	N	NDF	ADF	Lignin	Ash	Hemi	Mg	Mn	Na	P	S
		kg ha^−1^		%							mg kg^−1^				
**Forage mass**															
Native grass	Poultry litter	2588 ab^a^	46 a	44 a	1.62 a	61.27 a	34.31 a	7.25 a	1.28 a	26 a	1307 a	152 a	230 a	2478 a	1384 a
Native grass	Control	2326 ab	56 a	43 a	1.51 a	61.45 a	37.02 a	6.45 a	2.21 a	24 a	1429 a	141 a	139 a	2449 a	1437 a
Orchardgrass	Poultry litter	2312 ab	72 a	43 a	1.77 a	60.99 a	35.16 a	6.65 a	1.14 a	25 a	1868 a	114 a	382 a	3469 a	1987 a
Orchardgrass	Control	1218 b	58 a	44 a	1.85 a	60.59 a	34.12 a	6.60 a	1.39 a	26 a	1851 a	154 a	513 a	3079 a	2102 a
**Biomass accumulation**															
Native grass	Poultry litter	2588 a	42 a	44 a	1.67 a	63.90 a	33.62 a	5.98 a	1.18 a	30.18 a	1410 a	105 a	135 a	2912 ab	1474 b
Native grass	Control	2326 a	34 bc	45 a	1.48 a	64.79 a	33.83 a	5.41 a	1.33 a	30.82 a	1382 a	106 a	107 a	2308 b	1147 c
Orchardgrass	Poultry litter	2312 a	40 ab	44 a	1.83 a	61.26 a	33.74 a	7.74 a	1.27 a	27.58 a	1819 a	134 a	371 a	3731 a	2039 a
Orchardgrass	Control	1218 b	20 c	45 a	1.77 a	61.04 a	32.95 a	8.35 a	1.23 a	28.15 a	1734 a	143 a	506 a	3564 ab	2185 a

Forage mass and biomass accumulation and compositional parameters based on the two-way interaction of forage species [(C_4_; native grass mix, and C_3_; non-native, orchardgrass)] × fertility [fertilized with poultry litter and without)] averaged across sampling date (early-May, late-May, early-June, mid-June, and late-June] by harvest management.

^a^Different letters indicate a significant difference at *P* ≤ 0.05 within a given column [per forage parameter (forage mass and biomass accumulation)].

^b^*NDF* neutral detergent fiber, *ADF* acid detergent fiber, *Hemi*, hemicellulose, *C* carbon, *N* nitrogen, *Mn* manganese, *S* sulfur, *K* potassium, *P* phosphorus, *Mg* magnesium.

Percent PAR interception followed sigmoidal trends across all three years, whose functions included all 4 sampling dates (data not shown; DNS). The asymptotes corresponded to peak forage yields (forage mass and biomass accumulation), which occurred mid-June. No mathematical trends were observed for LAI across the grazing-season, but trends were similar to PAR interception (DNS). However, LAI tended to peak earlier; suggesting the canopy continued to effectively adsorb PAR despite the loss of chlorophyll lower in the canopy as the grazing-season progressed.

### Animal grazing intensity based on management and landscape

**Figure 1:**
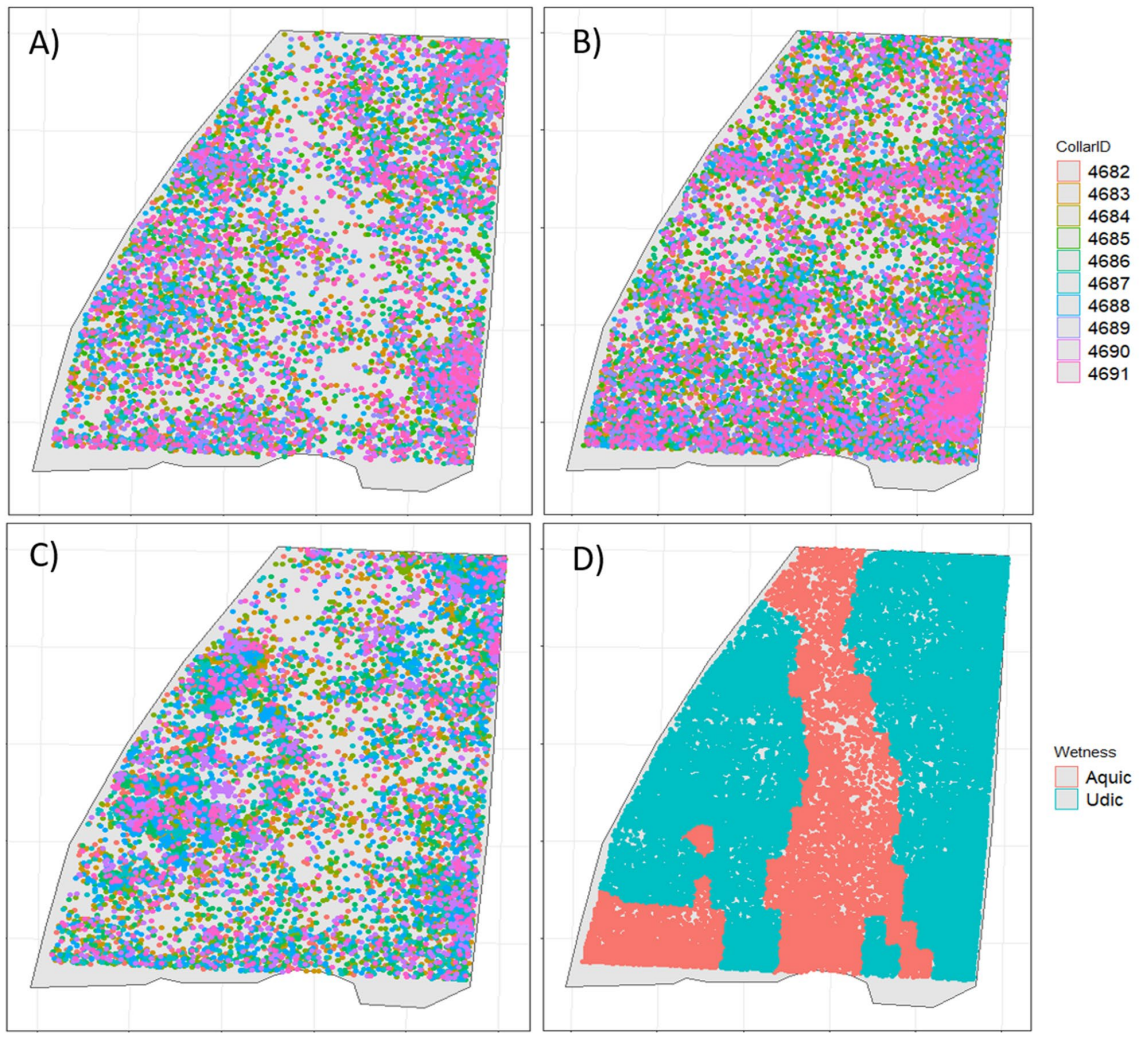
Cattle grazing preference linkage to soil wetness. Animal grazing frequency from 2017 to 2019 at a silvopastoral site in Fayetteville AR. All cattle collar GPS data recorded outside of the study area and/or with dilution of precision greater than four were removed, as were all tilt data (with tilt percentage less than 70% in the previous five minutes). Data were subsequently combined for fix and tilt and are displayed as individual animals grazing visits for 2017 (**A**); 2018 (**B**); and 2019 (**C**). Grazing data points with wet/aquic and dry/udic treatment (**D**) show spatial distribution of the treatment within the study site.

**Figure 2:**
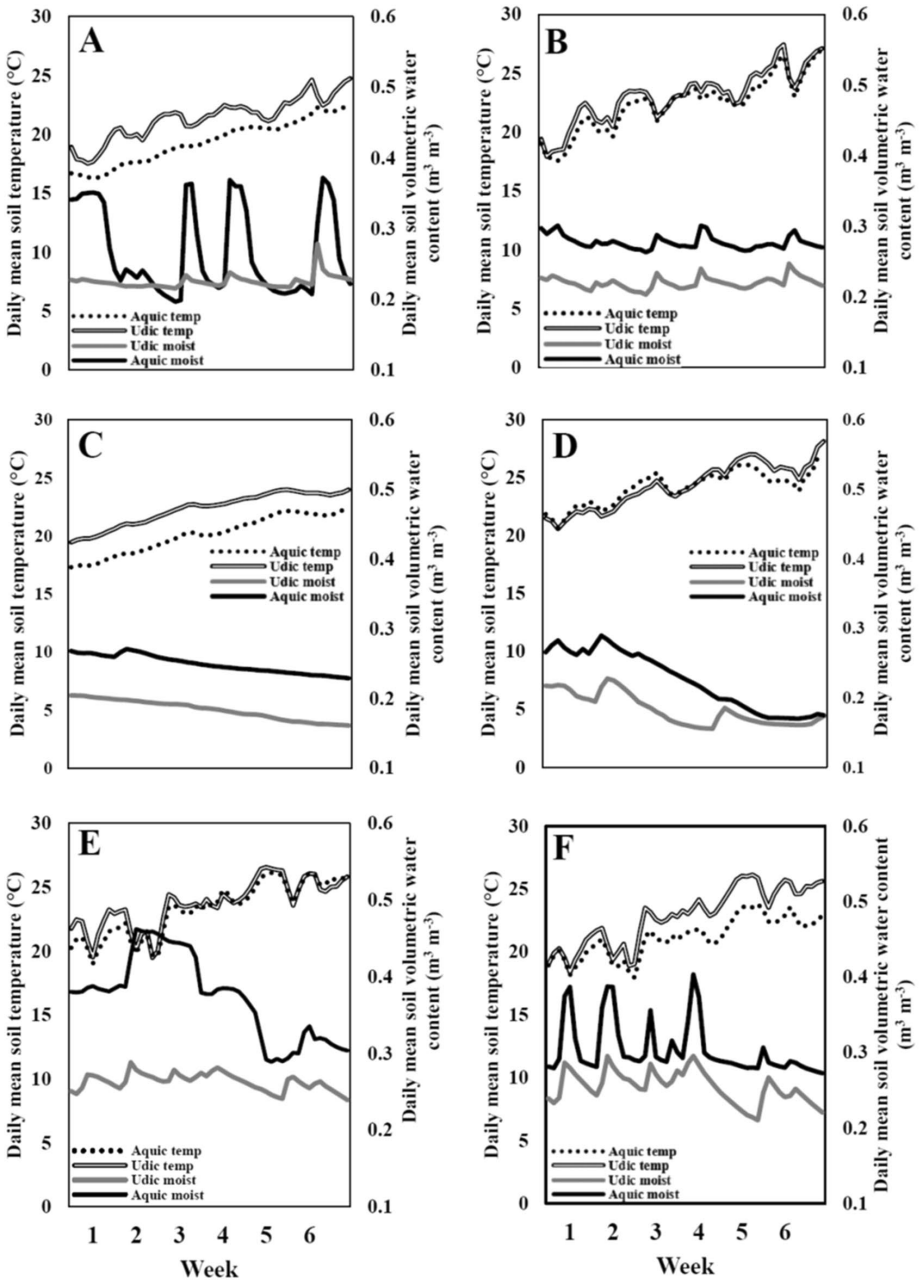
Daily volumetric soil water and temperature for aquic and udic treatments. Daily mean volumetric soil water content and daily mean soil temperature for the wet (aquic) and dry (udic) treatments from May–July, (**A**) 2017, at 15 cm; (**B**) 2017, 60–75 cm depth; (**C**) 2018, at 15 cm, and (**D**) 2018, 60–75 cm depth; (**E**) 2019, at 15 cm, and (**F**) 2019, 60–75 cm depth.

Animal grazing patterns varied spatially, temporally, and annually (Fig. 1). Overall, a lower number of animal grazing hours were spent in wetter, deeper soils (i.e. > 100 cm) across all years (Table 2; Fig. 1). Soil moisture regime patterns were fairly consistent across years, with distinct landscape-based patterns occurring (Fig. 2). Although in 2019 a somewhat different grazing pattern was observed (Fig. 1), even though aquic and udic treatments had a similar VWC, albeit only at the 15-cm depth during week 3 (Fig. 2).

**Table 2:** ANOVA of cattle grazing frequency.

	DF^a^	SS	MS	F value	Pr(> F)
Rep	2	164.436	82.218	4.166	0.023
Year	2	2534.196	1267.098	64.210	0.000
Week	8	658.540	82.318	4.171	0.001
Year:Week	10	805.471	80.547	4.082	0.001
Error (main plot)	39	769.611	19.734	–	–
Moisture	1	383.264	383.264	60.969	0.000
Year:moisture	2	397.426	198.713	31.611	0.000
Week:moisture	8	58.792	7.349	1.169	0.342
Year:week:moisture	10	171.797	17.180	2.733	0.012
Error (sub plot)	39	245.164	6.286	–	–
Spp	1	2093.290	2093.290	138.732	0.000
Moisture:Spp	1	10.666	10.666	0.707	0.404
Year:Spp	2	360.108	180.054	11.933	0.000
Week:Spp	8	212.223	26.528	1.758	0.103
Year:moisture:Spp	2	63.662	31.831	2.110	0.130
Error (sub-sub plot)	63	950.587	15.089	–	–
Fertility	1	0.006	0.006	0.000	0.987
Year:fertility	2	9.45	4.729	0.211	0.810
Week:fertility	8	66.44	8.305	0.370	0.935
Moisture:fertility	1	53.50	53.506	2.383	0.125
Spp:fertility	1	17.67	17.676	0.787	0.377
Year:week:fertility	10	177.12	17.712	0.789	0.639
Year:moisture:fertility	2	13.05	6.529	0.291	0.748
Year:Spp:fertility	2	8.52	4.261	0.190	0.827
Week:moisture:fertility	8	38.86	4.858	0.216	0.988
Week:Spp:fertility	8	80.14	10.018	0.446	0.891
Moisture:Spp:fertility	1	39.70	39.700	1.768	0.186
Year:week:moisture:fertility	8	31.73	3.967	0.177	0.994
Year:week:Spp:fertility	8	201.21	25.152	1.120	0.354
Year:week:moisture:Spp:fertility	8	68.32	8.541	0.380	0.929
Year:moisture:Spp:fertility	2	56.56	28.28	1.260	0.287
Error	130	2918.33	22.49	–	–

Analysis of variance of weekly animal grazing hours (hr ha^−1^ AU^−1^) based on forage species [big bluestem (native grass mix) and orchardgrass (non-native)] × soil moisture (aquic and udic) × fertility [fertilized with poultry litter and a control (0 kg N ha^−1^)]. A dataset with a total of 3951 observations representing animal visit frequency by each individual animals was used for this analysis.

^a^*Spp,* forage species (native and non-native); *moisture*, soil moisture (wet/aquic and dry/udic); *fert*, fertility (fertilized with poultry litter and a control); *week*, week of grazing-season, *DF* degrees of freedom, *SS* sum of squares, *MS* mean square error.

Animal visits or grazing frequency (hr ha^−1^ AU^−1^) across the landscape varied by week, year, soil moisture, and forage species (*P* ≤ 0.05), and year × week, year × soil moisture and year × forage species, but no three-way interaction occurred for any other factors except year × week × soil moisture (Table 2). Twenty nine percent greater grazing frequencies occurred in the udic soils (across forage species, grazing weeks, fertility treatments, and years). Similarly, cattle were 127% more likely to graze the native grass mix than the C_3_ species orchardgrass, which was also reflected in forage yields (Table 1).

**Figure 3:**
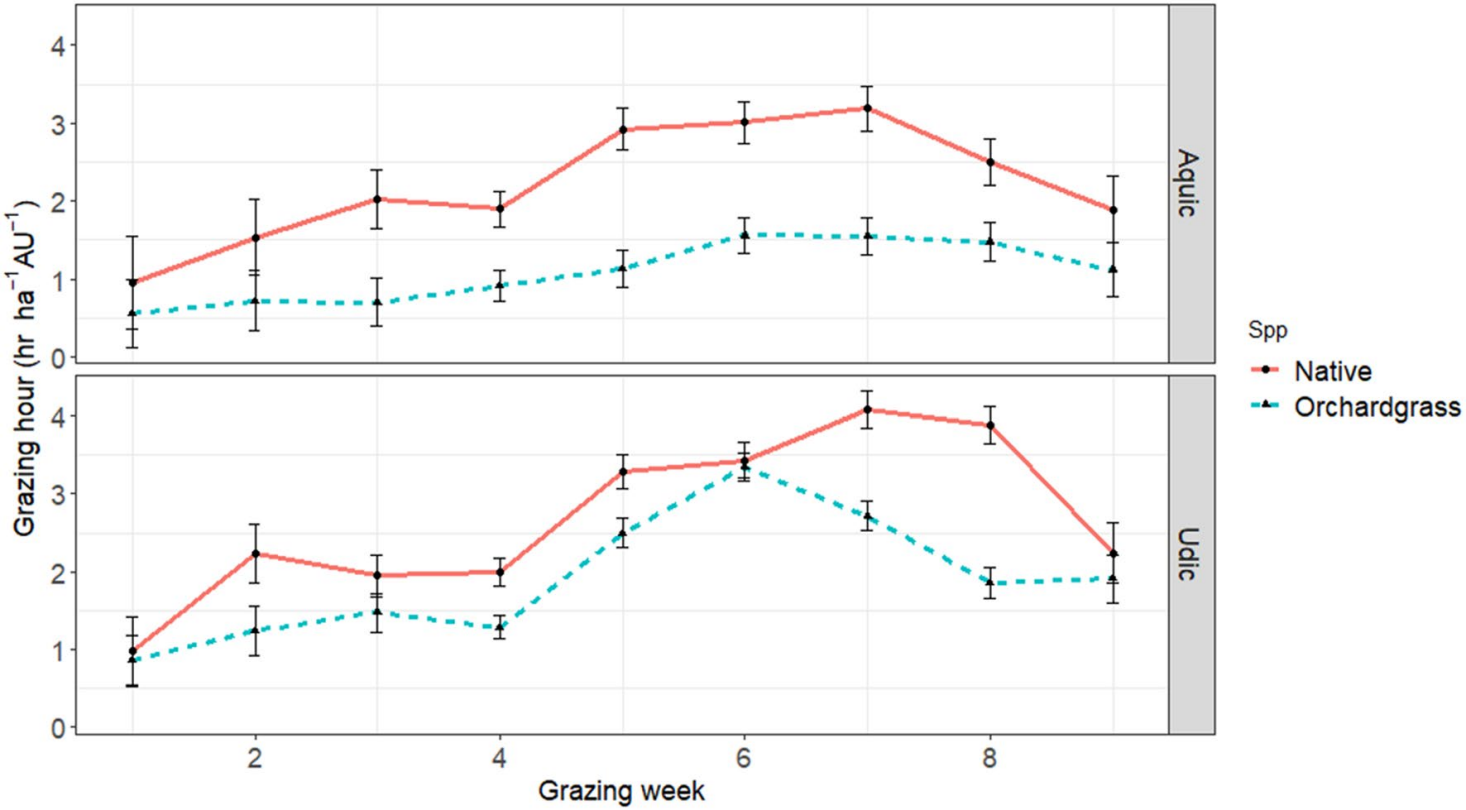
Animal grazing frequency per week. Animal grazing frequency (hr ha^−1^ AU^−1^) per week combined across years (2017–2019) based on forage species [big bluestem (native grass mix) and orchardgrass (non-native)] × soil moisture (wet/aquic and dry/udic) at a silvopastoral site in Fayetteville AR. Animal visits were averaged across fertility [fertilized with poultry litter and a control (0 kg N ha^−1^)] levels.

A year and moisture interaction (*P* ≤ 0.05) was also observed for weekly grazing hour. Overall, animal grazing frequencies (hr ha^−1^ AU^−1^) were 1.15, 1.77, and 3.32 for 2017, 2018, and 2019, respectively. Similarly, averaged across year, fertility, soil moisture, and forage species grazing hour increased steadily from week 1 to week 7 (0.81, 1.33, 1.51, 1.29, 2.16, 2.38, and 2.58 h ha^−1^ AU^−1^, similar to the trend shown in Fig. 3) and decreased afterwards (2.25 and 1.55 h ha^−1^ AU^−1^) for the remaining of the grazing period (weeks 8 and 9, respectively). Cattle preferred grazing the native grass mix compared to orchardgrass (Fig. 3) throughout the grazing period, except during the first week of the grazing period, where grazing preference was statistically not different between the two grass functional groups.

### Relationship between grazing and terrain and above-ground attributes

Grazing frequency was negatively correlated with only one terrain attribute, hillshade (Table 3). Hillshade index (relative measure of incident light on each cell). Additionally, hillshade was positively correlated with tree height and diameter (Table 3), indicating larger trees were associated with higher incidence of light and thus provided shade. Counter to expectations, among forage parameters, grazing hour (hr ha^−1^ AU^−1^) was negatively correlated with WSC content (r = − 0.75), accumulated biomass crude protein (r = − 0.44), and accumulated biomass lignin (r = − 0.65), while it was positively correlated with forage mass C (r = 0.51), forage mass lignin (r = 0.51), and accumulated biomass NDF content (r = 0.77).

**Table 3:** Systems-level correlation coefficient of cattle grazing pressure.

	Graze_hr^a^	WSC	PAR	LAI	Forage mass	Biomass accum	Tree Height	DBH	Forage mass NDF	Forage mass ADF	Forage mass C	Forage mass CP	Forage mass Lignin	Biom. accum NDF	Biom. accum. ADF	Biom. accum. C	Biom. accum. CP	Biom. accum. Lignin
	r*																	
Aspect^b^	− 0.35	0.03	0.06	0.01	0.11	− 0.38	0.36	**0.40**	− 0.16	− 0.02	− 0.26	0.08	− 0.26	− 0.02	− 0.24	0.00	0.08	0.15
Elevation	− 0.30	**0.46**	− 0.24	− 0.22	0.04	0.04	0.08	0.08	0.24	− 0.04	− **0.43**	0.04	− **0.43**	− 0.23	**0.71**	**0.50**	**0.50**	**0.48**
FlowAccum	0.27	− 0.16	− 0.05	− 0.04	− 0.08	− 0.09	0.15	0.07	− 0.16	− 0.25	0.22	− 0.09	0.22	0.17	− **0.43**	− 0.33	− 0.15	− 0.19
Hillshade	− **0.43**	− 0.07	− 0.03	− 0.10	− 0.06	− 0.21	**0.59**	**0.59**	− 0.08	− 0.24	− 0.05	0.16	− 0.05	0.13	− 0.23	− 0.06	0.10	− 0.01
LSFactor	0.15	0.04	− 0.15	− 0.08	0.07	0.18	− 0.37	− 0.32	0.28	**0.40**	− 0.16	− 0.19	− 0.16	− 0.05	**0.50**	**0.42**	− 0.05	0.07
MidSlope	0.31	− 0.33	− 0.16	− 0.16	0.02	− 0.08	0.33	0.33	0.02	− 0.12	0.36	0.00	0.36	0.23	− 0.26	− 0.12	− 0.10	− 0.27
MRRTF	− 0.15	0.03	0.49	**0.46**	0.18	− 0.06	0.11	0.09	0.11	0.00	− 0.07	0.05	− 0.07	− 0.18	− **0.41**	− 0.37	− 0.16	− 0.19
MRVBF	0.35	− 0.34	− 0.20	− 0.23	− 0.14	− 0.01	0.29	0.27	− 0.39	− 0.32	0.35	− 0.01	0.35	0.39	− **0.43**	− 0.31	− 0.09	− 0.27
NormHt	− 0.34	**0.40**	− 0.03	0.00	0.06	0.05	− 0.29	− 0.23	0.13	0.05	− 0.36	− 0.06	− 0.36	− 0.22	0.37	0.21	0.24	0.29
SAGAWI	0.19	− 0.22	0.04	0.01	− 0.03	− 0.07	0.32	0.23	− 0.17	− 0.24	0.20	− 0.01	0.20	0.17	− **0.48**	− 0.37	− 0.15	− 0.27
SlopeHt	− 0.32	0.35	− 0.01	0.04	0.14	0.04	− **0.44**	− **0.40**	0.13	0.17	− **0.44**	− 0.23	− **0.44**	− 0.18	0.34	0.21	0.05	0.18
SlopePer	0.04	0.18	− 0.04	0.01	0.17	0.07	− 0.37	− 0.33	0.30	0.33	− 0.20	− 0.14	− 0.20	− 0.21	**0.48**	0.34	0.12	0.20
soildepth	0.21	− 0.06	0.17	0.13	− 0.14	− 0.05	0.30	0.13	− 0.13	− 0.11	0.19	0.16	0.19	0.04	− 0.15	− 0.06	− 0.07	− 0.01
ValleyDep	0.19	− 0.28	0.02	0.08	0.20	− 0.10	− 0.13	− 0.29	0.20	**0.45**	0.09	− 0.36	0.09	0.11	− 0.04	0.09	− 0.37	− 0.35
VDistChn	− 0.31	**0.41**	− 0.14	− 0.11	0.13	− 0.06	− 0.01	0.07	0.15	− 0.05	− 0.35	0.02	− 0.35	− 0.19	**0.42**	0.31	0.35	0.35
Graze_hr	–	− **0.75**	− 0.10	− 0.19	− 0.13	0.34	− 0.06	0.05	− 0.28	0.20	**0.51**	− 0.36	**0.51**	**0.77**	− 0.14	0.01	− **0.44**	− **0.65**

Pearson correlation coefficient (r) of weekly animal grazing frequency (hr ha^−1^ AU^−1^) and terrain attributes with above-ground data [forage (forage mass and biomass accumulation) and tree parameters] using samples collected from 2017 to 2019.

^a^Graze_hr, animal visits (hr ha^−1^ AU^−1^); WSC,  forage water soluble carbohydrates (g kg^−1^ dry matter); *PAR* photosynthetically active radiation (µmol m^−2^ s^−1^), *LAI* leaf area index (index), forage mass,  forage collected from within enclosures every 10 days during the grazing season (kg ha^−1^); biomass accumulation,  total yield accumulated in grazed areas every 10 days during the grazing season (kg ha^−1^); *TreeHeight* tree height (m), *DBH* diameter breast height (m), *NDF* neutral detergent fiber (%), *ADF* acid detergent fiber (%), *C* carbon (%), *CP* crude protein (%).

^b^*Elevation* elevation (m), *Aspect* Aspect (°), *FlowAccum* flow accumulation (number of pixels, n), *LSFactor* slope-length factor (m), *MidSlope* mid-slope position (index), *MRRTF* multi-resolution ridge top flatness index (index), *MRVBF* multi-resolution valley bottom flatness index (index), *NormHt* normalized height (index), *SlopePer* slope percent (%), *SlopeHt* slope height (m), *SAGAWI* system for automated geoscientific analysis wetness index (index), *ValleyDep* valley depth (m), *VDistChn* altitude above channel network (m), *Hillshade* illumination value scaled to 0–255 considering the slope and aspect (index).

*r ≥ 0.40 are significant at *P* = 0.05 levels, hence bolded r values indicate significance.

The terrain attribute elevation had a positive correlation with WSC, accumulated biomass ADF, C, CP, and lignin, while it was negatively correlated with forage mass C, ADF, CP, and lignin (Table 3). This result indicates that more forage production was associated with higher elevation within the site (accumulated biomass). Some of other terrain attributes that were correlated with forage mass quality parameters were slope length factor (correlated with ADF), slope height (forage mass C and lignin) and valley depth (ADF). Accumulated biomass ADF was the forage parameter best correlated with several of the terrain attributes. It was positively correlated with elevation, slope length factor, slope percent, and altitude above channel network (VDistChn), while negatively correlated with flow accumulation, multi-resolution ridge top flatness index (MRRTF), valley bottom flatness index (MRVBF), and SAGAWI. LAI was positively related with only one terrain attribute, MRRTF, while PAR showed no relationship with any terrain attribute. Overall, higher elevation and relatively dry (udic) areas showed greater forage mass WSC and less lignin content, while lower elevation and wet areas showed less forage WSC content. Additionally, greater slope height differences (SlopeHt) resulted in negative tree height and DBH.

### Relationship between grazing and terrain and soil parameters

In general, sites with higher P (r = 0.51) and K (r = 0.66) content were associated with greater grazing frequency (hr ha^−1^ AU^−1^). Soil P and K were also negatively correlated with the terrain attribute ‘valley depth.’ Valley depth is calculated as vertical distance to channel network (of inverted DEM); hence it is a relative height difference between local ridges and a cell. Higher values indicate lower elevation (more difference with the ridges) within the site. A negative correlation of soil P and K with terrain attribute valley depth suggests that udic areas (higher elevation) were associated with higher P and K content and correspondingly these sites were preferred by cattle to graze (Table 4). With increasing aspect (direction of the steepest slope from the north), soil pH, C, N, and P decreased while bulk density increased (Table 4). With increasing flow accumulation (flowaccum) value (number of upland cells draining to a given cell, indicating lower elevation), soil bulk density decreased. Among soil parameters, soil pH and bulk density were the two parameters most correlated with several terrain attributes (Table 4).

**Table 4:** Correlations among cattle grazing pressure and terrain and soil parameters.

	Temp^a^	pH	EC	OM	C	N	CN	P	K	Sand	Silt	Clay	BD
	r*												
Aspect^b^	− 0.18	− **0.48**	− 0.34	− 0.36	− **0.53**	− **0.55**	− 0.03	− **0.46**	0.07	− 0.15	0.22	− 0.04	**0.76**
Elevation	0.30	− 0.07	0.21	0.02	0.19	0.17	− 0.08	0.16	− 0.20	0.34	− 0.33	− 0.09	0.20
FlowAccum	− 0.06	0.17	0.24	0.22	0.04	0.14	− 0.20	− 0.01	− 0.09	0.07	− 0.21	0.16	− **0.41**
Hillshade	− 0.12	− 0.25	0.00	− 0.03	− 0.20	− 0.11	− 0.32	− 0.06	0.14	0.05	0.03	− 0.12	**0.55**
LSFactor	0.26	− 0.06	0.01	− 0.12	0.05	− 0.04	0.27	0.03	− 0.18	− 0.11	− 0.01	0.17	− 0.11
MidSlope	− 0.17	0.35	0.36	0.06	0.00	0.09	− 0.29	0.02	0.14	0.06	0.03	− 0.13	− 0.20
MRRTF	− 0.09	− **0.40**	− 0.20	− 0.20	− 0.25	− 0.15	− 0.24	− 0.12	− 0.13	− 0.35	0.24	0.23	− 0.08
MRVBF	− 0.19	0.32	0.21	0.25	0.09	0.14	− 0.14	0.01	0.32	0.11	− 0.01	− 0.15	− 0.15
NormHt	0.28	− **0.44**	− 0.20	− 0.15	− 0.02	− 0.06	0.03	0.17	0.05	− 0.07	− 0.11	0.24	0.24
SAGAWI	− 0.21	0.19	0.19	0.19	0.03	0.10	− 0.15	− 0.09	0.04	− 0.02	0.07	− 0.05	− 0.27
SlopeHt	0.38	− **0.53**	− 0.22	− 0.25	− 0.14	− 0.19	0.09	− 0.01	− 0.07	− 0.28	0.01	0.41	0.11
SlopePer	0.17	− 0.07	− 0.05	− 0.14	0.05	− 0.02	0.20	0.07	− 0.14	− 0.05	0.00	0.07	− 0.16
soildepth	− 0.15	**0.51**	0.20	0.31	0.16	0.16	0.06	− 0.15	− 0.29	0.34	− 0.18	− 0.29	− 0.27
ValleyDep	0.14	0.18	0.01	− 0.28	− 0.38	− 0.35	− 0.03	− **0.50**	− **0.57**	− 0.34	0.24	0.21	− **0.45**
VDistChn	0.29	− **0.44**	− 0.03	− 0.20	− 0.10	− 0.07	− 0.21	0.22	0.14	0.04	− 0.17	0.16	0.38
graze_hr	0.06	0.11	0.01	0.26	0.31	0.34	− 0.02	**0.51**	**0.66**	− 0.35	0.30	0.15	0.18

Pearson correlation coefficient (r) of terrain attributes and weekly animal grazing frequency (hr ha^−1^ AU^−1^) with soil parameters using samples collected from 2017 to 2019.

^a^Temp,  soil temp at 0–15 cm (°C); *EC* electrical conductivity (dS m^−1^); *OM* organic matter (%),*C* carbon (%), *N* nitrogen (%), *CN* carbon:nitrogen (ratio), *P* phosphorus (mg kg^−1^), *K* potassium (mg kg^−1^), *BD* bulk density (kg m^−3^).

^b^*Elevation* elevation (m), *Aspect* Aspect (°), *FlowAccum* flow accumulation (number of pixels, n), *LSFactor* slope-length factor (m), *MidSlope* mid- slope position (index), *MRRTF* multi-resolution ridge top flatness index (index), *MRVBF* multi-resolution valley bottom flatness index (index), *NormHt* normalized height (index), *SlopePer* slope percent (%), *SlopeHt* slope height (m), *SAGAWI* system for automated geoscientific analysis wetness index (index), *ValleyDep* valley depth (m), *VDistChn* altitude above channel network (m), *Hillshade* illumination value scaled to 0–255 considering the slope and aspect (index), *Graze_hr* animal visits (hr ha^−1^ AU^−1^).

*r ≥ 0.40 are significant at *P* = 0.05 levels, hence bolded r values indicate significance.

### Grazing frequency visualized through radar chart

**Figure 4:**
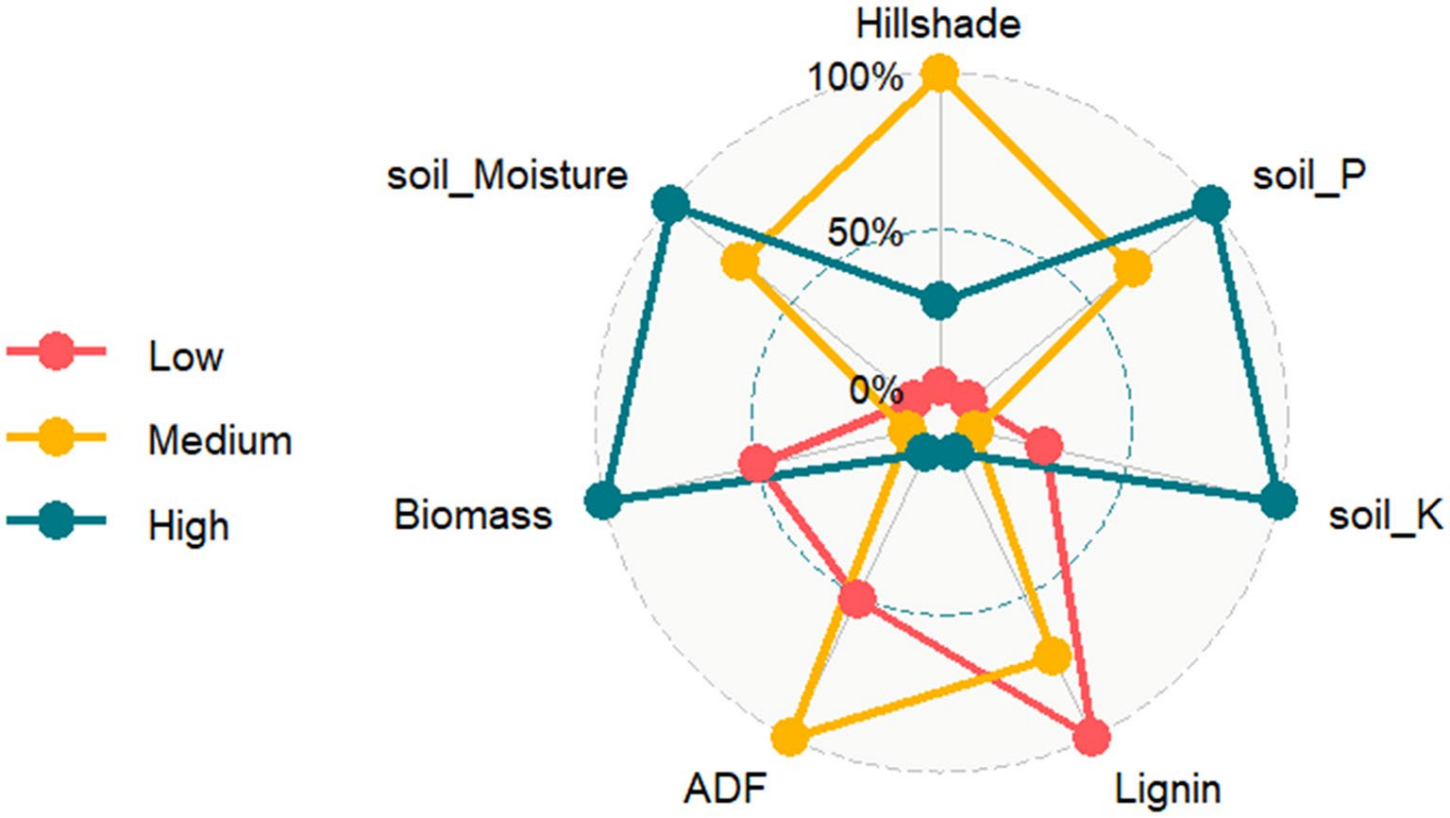
System-level linkages among grazing frequency. Radar chart illustrating indicators for low (< 1.30), medium (1.30–2.30), and high (> 2.30) weekly grazing frequency (hr ha^−1^ AU^−1^) with selected plant, soil, and terrain attribute variables from 2017 to 2019 at a silvopastoral site in Fayetteville AR based on cattle collar GPS data.

Factors most affecting grazing, including soil, terrain, and forage parameters were evaluated via a radar chart. Overall, high grazing frequencies were strongly related with soil moisture, soil P, soil K and accumulated biomass yield (Fig. 4). Although, the terrain attribute hillshade was most correlated with grazing frequency (hr ha^−1^ AU^−1^) at the medium frequency grazing level. On the other hand, lower grazing frequency was correlated with accumulated biomass lignin content. Therefore, animal grazing was responsive to environment, with higher grazing frequencies being linked to soil and plant parameters, medium grazing frequencies being driven by terrain, and lower grazing frequencies linked with plant composition (Fig. 4).

## Discussion

Forage mass was greatest for orchardgrass fertilized with poultry litter; however, it did not differ from the non-fertilized native grass mix, with the fertilized native grass mix and the non-fertilized orchardgrass being lowest. Therefore, poultry litter applications did not increase C_4_ forage species yields as it did with the C_3_ orchardgrass; which is likely owing to native warm-season grasses not having a high nutrient requirement^19,20^. Poultry litter applications for both forages resulted in the greatest N removal and P content. These minimal differences indicate the native and introduced forages have similar nutritive and compositional quality during the dates sampled and that poultry litter application rates resulted in little additional forage mass, composition, and nutritive gains (Table 1).

In order to maximize animal production efficiency in grazing systems, it is important to know herbage mass and whether livestock can effectively utilize the forage mass. Thus, rapid monitoring of pastures is needed, considering the use of traditional pasture monitoring tools to measure the quality and quantity of forage can be labor and time intensive^21,22^ and delays in results reduce the potential benefit to producers. Therefore, real-time data collection (e.g., NDVI) is needed for efficient management of pasture resources^23,24^. Future work will be done to evaluate NDVI linkages to cattle grazing patterns based on terrain attributes.

In general, heifers tended to favor non-aquic areas in the field, which is also visualized in Fig. 1. In another grazing behavioral study across landscapes using GPS tracking collars, ref.^18^ also identified grazing distribution linkages to environmental conditions, albeit in a range system. The present study is the only study that evaluates soil property linkages to plant growth and production and ultimately to grazing response based on terrain features.

Poultry litter fertilizer applications did not affect animal grazing spatially, likely owing to nominal rates being applied with roughly only 50% N in poultry litter being plant available in year 1^20^. Finally, across all years, grazing frequencies were greatest during weeks 6 and 7, perhaps owing to increased dietary reproduction needs and less overall forage mass available, thus requiring more grazing to obtain adequate nutrition (particularly for the C_4_ mix). Overall, cattle spent more grazing hours foraging the native grass species under dry soil moisture conditions, particularly as the season progressed, likely owing to the C_3_ species increasing in maturity and decreasing in palatability (Fig. 3). These results can be used to tailor grazing management decisions (where and how often), forage quality (via several metrics) and micro-climate (shade on hot days in hot environments).

Animals tend to prefer grazing forages that are more digestible and greater in macronutrients^25^. Our result indicates that after the first week, cattle selected the native grass species over orchardgrass. Animal grazing behavior studies have shown that cattle try to maximize either intake rates^26^ by preferring forages with more desirable nutritive value and physical characteristics^27,28^ or by selecting grazing conditions such as avoiding wet areas. Grazing preference of cattle in dry areas may be due to their mouth morphology^29–31^ and how cattle use their tongue to forage. One theory is that the lubricity of the moist leaf laminae may increase slippage between the incisors and dental pads; hence cattle may avoid grazing aquic areas, which is consistent with this study. In addition, it is conceivable that cattle simply prefer to not graze areas with wetter soils because wet hooves are not desirable.

Reference^9^ used terrain attributes of this site to develop topographical functional units (TFU). Authors observed that the TFU that represented lower elevation and aquic areas of the site were highest in soil element concentrations, while TFUs that represent udic areas (higher elevation) were lower in element concentration. Contrary to that finding, our study showed soil P and K were more associated with dryer parts of the study site (Table 4). Overall, topography parameters helped explain soil conditions, plant growth (tree and forages), and ultimately animal response across this continuum.

Grazing frequency was negatively correlated with the ‘hillshade’ parameter, indicating cattle preferred grazing in more shaded areas during the summer and that cattle avoided more illuminated areas spatially. Additionally, soil P and K were greater in udic areas and cattle preferred to graze on these areas spatially and temporally. Therefore, selected terrain attributes may be valid for predicting animal grazing preference dynamics in silvopasture systems. Overall, topography parameters helped explain soil properties—plant growth (tree and forages)—and ultimately animal grazing pressure across this continuum.

Degraded pasture systems are likely to result in the expansion of new land to fulfill carrying capacity requirements, which would increase anthropogenic pressure on pasture systems. This spatial monitoring approach holistically demonstrates these relationships temporally and spatially via remotely sensed tools. Results from this study could be scaled up for novel applications to improve grazing management in the largest land-use category in the U.S., grasslands, which would allow for sustainable intensification of forage-based livestock production to meet growing protein demands and expectations for environmentally responsible production. Therefore, spatial monitoring technologies can be used to tease apart factors underpinning plant-soil–water linkages via a systems-level evaluation for optimized grassland agroecosystems.

## Materials and methods

### Site description

**Figure 5:**
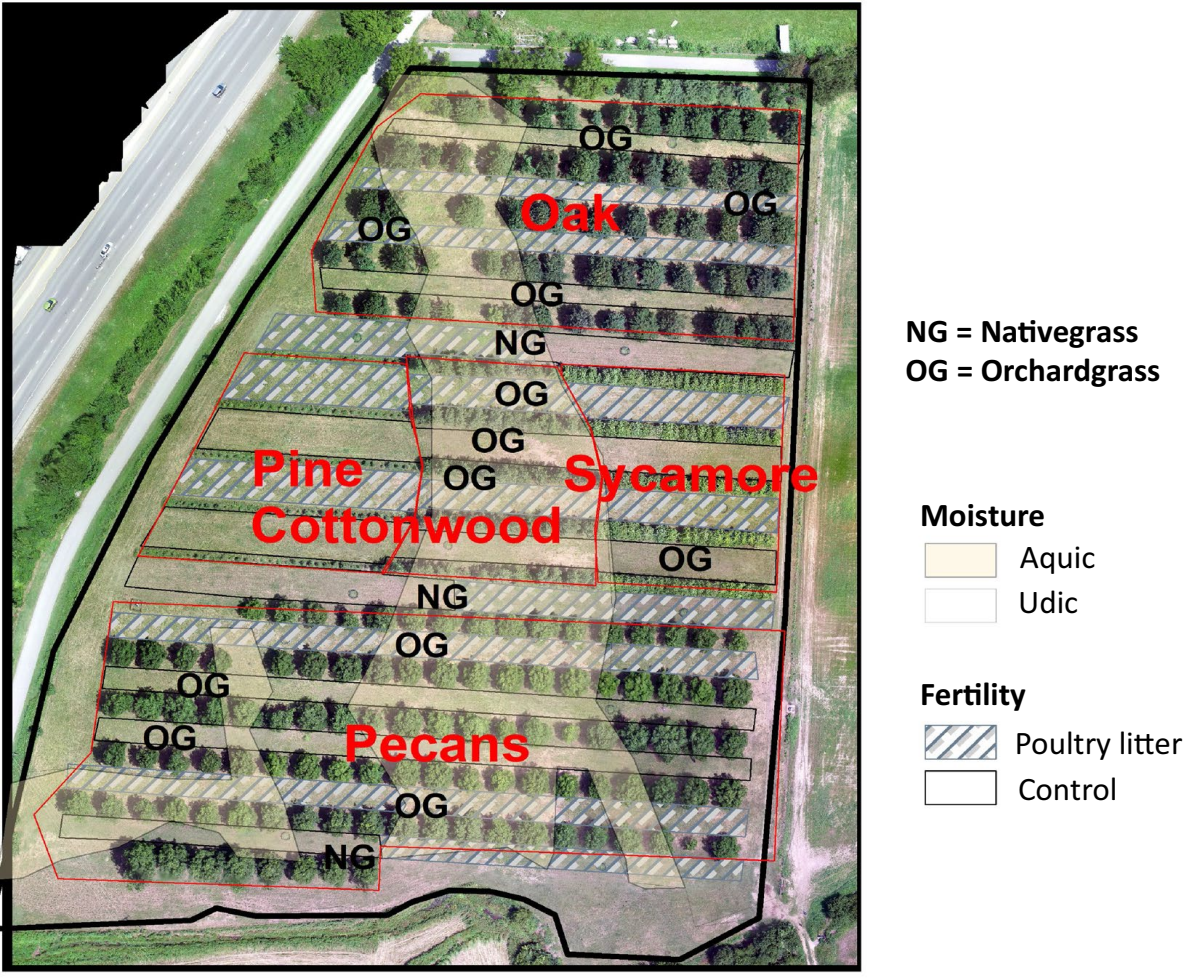
Study site with tree species labels (oak, pine, cottonwood, sycamore, and pecans) and area representing the individual tree polygons. Tree polygons extend southbound to identify grass species treatments (planted in tree alleys) associated with each individual tree. Imaging was collected using DJI- Inspire 1-Zenmus X3 camera (RGB) mounted on Drone and DroneDeploy (https://www.dronedeploy.com/) and was processed and exported using the orthomosaic output.

This study was conducted on a 4.25-ha paddock (Fig. 5) located at the University of Arkansas Agricultural Research and Extension Center in Fayetteville, AR (36.09° N, 94.19° W). The site is located in the Ozark Highlands, Major Land Resource Area 116A^32^. Information on previous site history is described by ref.^33^. Briefly, soil in most of the experimental area is mapped as Captina silt loam (fine-silty, siliceous, active, mesic Typic Fragiudults) with some Pickwick silt loam (fine-silty, mixed, semiactive, thermic Typic Paleudults) and small areas of Johnsburg silt loam (fine-silty, mixed, active, mesic Aquic Fragiudults), and Nixa cherty silt loam (loamy-skeletal, siliceous, active, mesic Glossic Fragiudults^34^). The field also contains a dissimilar inclusion that is lower in elevation and was not captured in the mapping unit. The wetter location within that this site is classified as fine, mixed, active, thermic Typic Endoaqualfs and is termed an “aquic” soil moisture regime^33^.

During tree establishment in 2000, sixteen rows of three species including northern red oak (*Quercus rubra* L.), eastern black walnut (*Juglans nigra* L.), and pecan [*Carya illinoinensis* (Wangenh). K. Koch] were oriented east–west with 15-m spacing between rows. In 2014, the rows of eastern black walnut trees were each replaced with three species: American sycamore (*Platanus occidentalis* L.), cottonwood (*Populus deltoides* W. Bartram ex Marshall), and pitch/loblolly hybrid pine (*Pinus rigida* × *P. taeda*). Two forage species treatments were established in the alleys between tree rows, including a cool-season species [orchardgrass (*Dactylis glomerata* L., var. Tekapo)] which was seeded fall 2015 at 17 kg pure live seed (PLS) ha^−1^ and a native warm-season mix [8:1:1 big bluestem (*Andropogon gerardii* Vitman), little bluestem (*Schizachyrium scoparium* {Michx. Nash} and indiangrass (*Sorghastrum nutans* L.)], seeded spring of 2016 at 10 kg PLS ha^−1^. Alleys were planted with a Haybuster 107C no-till drill (DuraTech, Jamestown, ND) and replicated thrice. Prior to establishment, Cornerstone® Plus (N-[phosphonomethyl] glycine) was used at a 2.2 kg ha^−1^ rate (41% a.i.) to kill existing vegetation. After establishment, alleys were treated with Plateau (ammonium salt of imazapic) at 0.28 kg ha^−1^ rate (23.6% a.i.). The site receives an average (30-yr mean) annual precipitation of 1232 mm and has an average ambient temperature of 14.5 °C^35,36^.

### Treatment implementation and field management

Independent or explanatory variables included forage species (C_4_ native grass mixture and non-native C_3_ orchardgrass), fertility [poultry litter and an un-fertilized control (0 kg N ha^−1^)], and soil moisture regime in order to assess mechanisms for grazing preference. The primary treatment, or forage species, was implemented as described above with three replications. The second main effect, or fertility, was executed by forage treatments receiving locally-sourced poultry litter applied at a rate of 84 kg N ha^−1^ on March 21, 2017; March 21, 2018; and, April 12, 2019 (4.94 Mg ha^−1^, fresh weight basis). Chemical composition of poultry litter was 2.69%, 0.70%, 1.12%, and 6.1 for N, P, K, and pH, respectively, during 2017; 1.98%, 0.58%, 1.02%, and 6.2 for N, P, K, and pH, respectively, during 2018; and, 2.48%, 0.69%, 0.94%, and 5.2 for N, P, K, and pH, respectively, during 2019 (Arkansas Diagnostic Laboratory, Fayetteville, AR). The control fertility rate was represented by 0 N kg ha^−1^. The third main effect, or soil moisture regime, was documented by random placement of volumetric water content (VWC) sensors at two depths (15-cm and 60–75-cm) from the soil surface (Fig. 2). Water content and soil temperature measurements were recorded every 4 h throughout the experimental period from May to July in 2017–2019 and logged on a Decagon EM50 data logger (METER Group, Pullman, WA). Soil moisture data were averaged each day and expressed as daily mean VWC for further analysis (Fig. 2). Weather variables were measured by a micro-meteorological weather station approximately 500 m from the experimental site.

Ten Angus crossbred heifers (*Bos taurus*) freely grazed the site at a rate of 2.20 animal units (AU) ha^−1^ from May 11 to June 23 in 2017; 2.20 AU ha^−1^ from May 24 to July 6 in 2018; and, in 2019 2.42 AU ha^−1^ from May 29 to July 11. In 2017, heifers were weighed at 3503 kg initially, and 3800 kg when removed. In 2018, heifers again were weighed at 4012 kg initially and removed without weighing. In 2019, heifers weighed 4420 kg and 4632 kg when removed. A beef mineral was freely available during the grazing period. No other feed supplement was given. Ten GPS collars (Model 3300LR, Lotek Wireless Inc., Newmarket, ON) were fitted on heifers May 8, 2017, May 21, 2018, and May 27, 2019 and heifers were observed for 24 h prior to entering the silvopasture site. The georeferenced location was within 1 m accuracy and points where the collar was at a downward angle (indicating grazing) were recorded.

Photosynthetically active radiation (PAR) and leaf area index (LAI) of the forages were measured every 10 to 15 days using an AccuPAR LP-80 ceptometer (METER, Pullman, WA) throughout the study period (after grazing commenced). Approximately 30 measurements of PAR were made above and directly below the canopy in each forage experimental unit. Light measurements were recorded between 1000 and 1400 h local time. The light quantum sensor makes radiation measurements with an optical sensor by determining light interception centered on five zenith angles 7°, 23°, 38°, 53°, and 68°^37^, from which PAR interception by the canopy was computed. Latitude and longitude coordinates and heights were also recorded. In 2017, PAR and LAI were measured and manually calculated for shady and non-shady areas. For 2018 and 2019, LAI was calculated directly on the LP-80 ceptometer.

### Sample processing and compositional and chemical analyses

Soil samples per experimental unit (species, fertility, and soil moisture regime) were collected in triplicate on March 20, 2017, March 16, 2018, and May 17, 2019 at the 0- to 15-cm depth using a 2-cm push probe and composited. Plant material was manually removed and samples were dried in a forced-air oven at 70 °C for 48 h. Samples were subsequently ground and sieved to pass a 2-mm mesh. A modified hydrometer method was used to determine soil texture^38^. Additionally, total C and N were determined via combustion using a VarioMax CN analyzer (Elementar Americas, Mt. Laurel, NJ). Mehlich-3 extractable soil element concentrations were determined using a 1:10 soil mass:extractant solution volume ratio^39^ and analyzed by inductively coupled argon-plasma spectrometry (ICP, Agilent Technologies, Santa Clara, CA). Weight-loss-on-ignition was used to determine soil organic matter (OM) concentration after 2 h at 360 °C^40^. Soil pH and EC were measured on a subsample of the 1:10 (soil:water) sample extraction^41^. Bulk density (rb, g cm^–3^) was measured on a per plot basis using the Core Method with a 4.8-cm diameter core^42^.

Forage mass and accumulation samples thereof were collected throughout the summer grazing periods. For accumulation samples, 4-m^2^ cattle exclosures (three per species and fertility combination) were placed and secured in alley centers to minimize shading effects^43,44^. Sampling occurred on 3 sampling dates in 2017 (May 5, May 26, and June 16); 4 sampling dates in 2018 (May 25, June 4, June 13, and June 22), and 4 dates in 2019 (June 10, June 20, July 1, and July 11)^43^. During those sampling dates, three 0.25-m^2^ samples were collected from within exclosures (biomass accumulation) along with three similar samples from a nearby grazed area (forage mass). Both forage sets (forage mass and biomass accumulation) were clipped at 6 cm above-ground, geo-referenced, weighed, then dried at 70 °C for 48 h and reweighed to determine moisture content. After drying, samples were ground using a Wiley mill (Thomas Scientific, Swedesboro, NJ) to pass through a 1-mm screen. Total C and N were determined via high-temperature combustion using a VarioMax C:N analyzer (Elementar Americas, Mt. Laurel, NJ). Lignin, acid-detergent fiber (ADF), and neutral detergent fiber (NDF) were determined using an ANKOM 2000 Fiber Analyzer (ANKOM Technologies, Macedon, NY^45^). Hemicellulose was calculated by ADF minus NDF; crude protein by multiplying percent N by 6.25; and nutrient (N, P, K) removal by multiplying nutrient^46^ (OES DV (Perkin-Elmer, Waltham, MA). Total ash was determined based on ASTM standard E1755-01^47^. One gram of ground, prepared forage tissue (sieved to 1 mm) was placed in an oven-dried, porcelain crucible overnight at 105 °C. Crucibles were placed in a muffle furnace at 575 °C for 4 h. After 4.5 h from the start of furnace, crucibles were removed and cooled to room temperature in a glass desiccator. The material retained in the crucible was weighed and ash concentration was expressed as g kg^−1^.

Water soluble carbohydrate (WSC) concentration of accumulated biomass (ungrazed tissue) was measured using a calorimetric procedure described by ref.^43^. Briefly, per ref.^48^ standards were prepared by mixing 0.1 g of dextrose with 250 mL distilled water, then 0.25 g of each forage sample was soaked in distilled water for 2 h and the solution was filtered. Samples and standards were transferred to glass tubes and 0.13 mL of 0. 90% (wt/wt) phenol and 5 mL of concentrated H_2_SO_4_ was added. Samples were then placed at room temperature for 10 min followed by incubation for 20 min in a water bath at 28 °C. Absorbance was measured on a spectrophotometer (SPECTRAmax 250, Molecular Products, Sunnyvale, CA) set to 490 nm wavelength.

Tree diameter at breast height (DBH; 137 cm above soil level) was collected during the dormant season from 2017 to 2019 (4 sampling dates) and were used to represent tree diameter during grazing periods and can be referenced in^49^. On each sampling date, a single measurement was taken per tree. During the study period, 215 observations for cottonwood, 213 for oak, 385 for pecan, 269 for pine, and 234 observations for sycamore were used as a proxy for tree biomass and canopy coverage. Tree heights (2017 to 2019) were also used in the correlation analysis. During this period, 215, 213, 290, 509, and 235 tree height observations were collected for cottonwood, oak, pecan, pine, and sycamore, respectively.

All methods were carried out in accordance with relevant guidelines and regulations. All experimental protocols were approved by the Institutional Animal Care and Use Committee at the University of Arkansas. Finally, the use of plants in the present study complies with international, national, and institutional guidelines and is in compliance with the IUCN Policy on Research Involving Species at risk of Extinction and the Convention on the Trade in Endangered Species of Wild Fauna and Flora.

### Forage and telemetry and terrain analysis and model development

Multivariate analysis of variance (MANOVA) tests of explanatory variables including forage quantity (forage mass and biomass accumulation) and nutritive/compositional variables (ADF, NDF, lignin, hemicellulose, ash, C, N, N removal, and minerals) were performed using the MIXED procedure of SAS (SAS V9.3; SAS Inst., Cary, NC). In this model, forage species, fertility (poultry litter and the control), soil moisture regime (aquic and udic), and sample date were considered fixed effects. Year and replication were entered as random effects using PROC MIXED. When effects or interaction confluences were found, mean separations were performed using the SAS macro ‘pdmix800′^50^ with Fisher’s least significant difference (LSD) at a Type I error rate of 5%^51^.

### Terrain attribute extraction from digital elevation model

For terrain and grazing data extraction, polygons were created to represent alleys within tree rows for each treatment combination^52^. Grazing and terrain values within the alley polygon (excluding tree canopy area) were extracted for further analysis. For terrain value extraction, grazing data points were overlaid on top of a 1 × 1 m terrain attribute raster developed for the study site^9^ and raster values were extracted for each grazing data point. Median raster value per experimental unit that represent the terrain attribute were prepared for correlation analysis between grazing and terrain attributes. A similar raster extraction method was used for soil, forage, and tree data for their respective correlation analysis. Terrain attributes used to describe landscape features included elevation, aspect (direction of steepest slope from the north), flow accumulation (FlowAccum; number of upland cells draining to a given cell), slope length factor (LSFactor; slope length used in universal soil loss equation), mid slope position (MidSlope; classification of slope position in valley and crest direction), multi resolution ridge top flatness index (MRRTF; high flat area), multi resolution valley bottom flatness index (MRVBF, flat valley bottom area), normalized height (NormHt; elevation scaled to 0–1), slope percent (SlopePer; maximum rate of change between a cell and its neighbors), slope height (SlopeHt; a relative height difference to the immediate adjacent crest line), system for automated geoscientific analysis wetness index (SAGAWI; a slope and specific catchment area based wetness index), valley depth (ValleyDep; a relative height difference to the immediate adjacent channel lines of inverted DEM), altitude above channel network (VDistChn; difference between channel base and surface elevation), and hillshade (Hillshade; illumination value scaled to 0–255 based on the slope and aspect).

Grazing data points with dilution of precision greater than four and tilt data with a percentage less than 70% in the previous five minutes were removed as per the instruction in the 3300 Lotek manual. Data were subsequently combined for fix and tilt. Data points that were outside the study boundary were also removed. Overall, raw data totaled 115,854 fixes with 36,019 being used. GPS coordinates were excluded if they: represented cattle loafing (non-grazing), had poor GPS quality (number and precision of satellite signals e.g., < 4 satellites per datum^52^), or were outside the grazing area (e.g., near watering system).

Animal grazing data points that were recorded every 15 min (if a cattle head was down > 70% of the time within a 15-min interval) were counted for each experimental unit during the grazing period as “animal visit” to that specific experimental unit. Animal visits were then converted to total hours per week for each grazing animal based on 15 min duration per observation. Since total areal coverage of different treatments were not equal (e.g., aquic vs. udic total area) and animals used within the study were different (10 heifers in 2017 and 2018 and 8 heifers in 2019), weekly grazing hours were then weighted based on the area associated with each treatment combination and expressed as animal grazing hour per hectare per animal units (hr ha^−1^ AU^−1^) for further analysis. A final dataset with 3951 observations that represent animal visit frequency per unique animal ID (GPS collar ID) over the grazing period (weekly) was used for the analysis.

Analysis of variance (ANOVA) was conducted on both weekly and total animal grazing frequency across years (hr ha^−1^ AU^−1^) using hierarchical design methods where year and weeks were the main factors; and, soil moisture, grass species, and fertility were the subplot, sub-subplot, and sub-sub-subplot (experimental unit), respectively. Each experimental units were replicated three times. A customized R^53^ code was developed based on ref.^49^ and ‘agricolae’ packages^54^ for ANOVA. Grazing hours and terrain attributes were then summarized for each treatment combination to explore the relationships among forage, soil, landscape (terrain), and tree parameters using Pearson correlation coefficient values at alpha level of 0.05. Based on correlation output, select variables were further explored using a radar chart where weekly grazing frequency were classified into low (< 1.30), medium (1.30–2.30), and high (> 2.30) grazing frequency (hr ha^−1^ AU^−1^) to visualize relationships between grazing and significant variables from the aforementioned analyses.
